# Correction: Non-linear links between human capital, educational inequality and income inequality, evidence from China

**DOI:** 10.1371/journal.pone.0316746

**Published:** 2024-12-30

**Authors:** Mo Xu, Shifeng Chen, Jian Chen, Taiming Zhang

There is an error in affiliation 3 for author Jian Chen. The correct affiliation 3 is: Department of Geography, Faculty of Social and Historical Sciences, University College London, London, United Kingdom.
