# Supplementary material for: Non-linear links between human capital, educational inequality and income inequality, evidence from China
Source: PLoS One. 2023 Aug 4;18(8):e0288966. doi: 10.1371/journal.pone.0288966 (PMC10403140; doi:10.1371/journal.pone.0288966)
Supplement: Attachment — Submitted filename: Comments on Non-linear relationship between human capital, educational inequality and income inequality, evidence from China.docx [file pone.0288966.s001.docx]

Comments on the revised manuscript: Non-linear relationship between human capital, educational inequality and income inequality, evidence from China.

Dear Dr. Muhammad Tayyab Sohail

Academic Editor

PLOS ONE

Thank you for giving me the opportunity to read and review this manuscript. I have the following comments:

- It is necessary to explain the justifications that were invoked when defining the model variables. By referring to the theoretical framework that confuses these variables, or by referring to the empirical literature in this context. Therefore, the authors should give clear justifications for choosing the study variables.
- The study uses the NARDL model to capture the nonlinear relationship with NARDL (Shin et al., 2014). Although using NARDL developed by (Shin et al., 2014) is a suitable technique for capturing the asymmetric effects of independent variables on a specific dependent variable, however, I think that this technique is more suitable if independent variables are characterized by volatility (increase and decrease), for example, oil prices or financial data. But this is not realized in the case of this study. This model requires observable volatility in the independent variables. The authors need to strengthen their arguments for choosing this model.
- On the other hand, there are many developments since the traditional linear ARDL model of (Pesaran et al., 2001) to capture asymmetric cointegration such as the QARDL model provided by (Cho et al., 2015) or dynamic ARDL (Jordan & Philips, 2018). Therefore, I think that the methodological choice should be better defended.
- This study uses annual time series data from 1975 to 2020. I believe that during this period structural changes occurred in the Chinese economy, which naturally affect the behavior of the variables included in the study. Thus, it is appropriate to test the stability of the time series using some tests that take structural breaks into account to determine the degree of integration of the time series more accurately (e.g. Clemente et al., 1998; Lee & Strazicich, 2003)

**General comment**: I feel that considering the above notes will improve the manuscript to be more rigorous.

Cho, J. S., Kim, T., & Shin, Y. (2015). Quantile cointegration in the autoregressive distributed-lag modeling framework. *Journal of Econometrics*, *188*(1), 281–300. https://doi.org/https://doi.org/10.1016/j.jeconom.2015.05.003

Clemente, J., Montañés, A., & Reyes, M. (1998). Testing for a unit root in variables with a double change in the mean. *Economics Letters*, *59*(2), 175–182.

Jordan, S., & Philips, A. Q. (2018). Cointegration testing and dynamic simulations of autoregressive distributed lag models. *The Stata Journal*, *18*(4), 902–923.

Lee, J., & Strazicich, M. C. (2003). Minimum Lagrange multiplier unit root test with two structural breaks. *Review of Economics and Statistics*, *85*(4), 1082–1089.

Pesaran, M. H., Shin, Y., & Smith, R. J. (2001). Bounds testing approaches to the analysis of level relationships. *Journal of Applied Econometrics*, *16*(3), 289–326.

Shin, Y., Yu, B., & Greenwood-Nimmo, M. (2014). Modelling asymmetric cointegration and dynamic multipliers in a nonlinear ARDL framework. In *Festschrift in honor of Peter Schmidt* (pp. 281–314). Springer.
